# Case report: atypical Silver-Russell syndrome patient with hand dystonia: the valuable support of the consensus statement to the wide syndromic spectrum

**DOI:** 10.3389/fgene.2023.1198821

**Published:** 2023-07-17

**Authors:** Alessandro Vimercati, Pierpaola Tannorella, Eleonora Orlandini, Luciano Calzari, Mirella Moro, Sara Guzzetti, Angelo Selicorni, Milena Crippa, Lidia Larizza, Maria Teresa Bonati, Silvia Russo

**Affiliations:** ^1^ Research Laboratory of Medical Cytogenetics and Molecular Genetics, IRCCS Istituto Auxologico Italiano, Milano, Italy; ^2^ Specialty School of Pediatrics, Alma Mater University of Bologna, Bologna, Italy; ^3^ Bioinformatics and Statistical Genomics Unit, IRCCS Istituto Auxologico Italiano, Milano, Italy; ^4^ Department of Endocrine and Metabolic Diseases and Lab of Endocrine and Metabolic Research, IRCCS Istituto Auxologico Italiano, Milano, Italy; ^5^ UOC Pediatria, ASST Lariana, Como, Italy; ^6^ Unit of Medical Genetics, Institute for Maternal and Child Health Istituto di Ricovero e Cura a Carattere Scientifico (IRCCS) Burlo Garofalo, Trieste, Italy

**Keywords:** Silver-Russell syndrome, unexpected molecular results, body asymmetry, IGF2, hand dystonia, VPS16, endolysosomal pathway

## Abstract

The amount of Insulin Growth Factor 2 (IGF2) controls the rate of embryonal and postnatal growth. The *IGF2* and adjacent *H19* are the imprinted genes of the telomeric cluster in the 11p15 chromosomal region regulated by differentially methylated regions (DMRs) or imprinting centers (ICs): H19/IGF2:IG-DMR (IC1). Dysregulation due to IC1 Loss-of-Methylation (LoM) or Gain-of-Methyaltion (GoM) causes Silver–Russell syndrome (SRS) or Beckwith–Wiedemann syndrome (BWS) disorders associated with growth retardation or overgrowth, respectively. Specific features define each of the two syndromes, but isolated asymmetry is a common cardinal feature, which is considered sufficient for a diagnosis in the BWS spectrum. Here, we report the case of a girl with right body asymmetry, which suggested BWS spectrum. Later, BWS/SRS molecular analysis identified IC1_LoM revealing the discrepant diagnosis of SRS. A clinical re-evaluation identified a relative macrocephaly and previously unidentified growth rate at lower limits of normal at birth, feeding difficulties, and asymmetry. Interestingly, and never previously described in IC1_LoM SRS patients, since the age of 16, she has developed hand-writer’s cramps, depression, and bipolar disorder. Trio-WES identified a *VPS16* heterozygous variant [NM_022575.4:c.2185C>G:p.Leu729Val] inherited from her healthy mother. VPS16 is involved in the endolysosomal system, and its dysregulation is linked to autosomal dominant dystonia with incomplete penetrance and variable expressivity. IGF2 involvement in the lysosomal pathway led us to speculate that the neurological phenotype of the proband might be triggered by the concurrent IGF2 deficit and *VPS16* alteration.

## 1 Introduction

Silver–Russell syndrome (SRS, MIM #180860) is a rare imprinting disorder (1:30.000–1:100.000), characterized by severe prenatal and postnatal growth retardation, relative macrocephaly at birth, body asymmetry, prominent forehead, and feeding difficulties. Clinical diagnosis is based on the occurrence of at least four out of six clinical signs, in accordance with the Netchine–Harbison clinical scoring system (NH-CSS), but molecular testing is recommended in patients with ≥3/6 criteria. The most common underlying mechanisms are loss of methylation of the paternal allele at *H19/IGF2:IG-DMR* in the 11p15.5 region (IC1_LoM and 30%–60% of cases) and maternal uniparental disomy of chromosome 7 (UPD(7)mat and 5%–10% of cases) involving *GRB10:alt-TSS-DMR*, *PEG10:TSS-DMR*, and *MEST:alt-TSS-DMR*. Other rare abnormalities, such as UPD(14)mat, UPD(16)mat, UPD(20)mat, and pathogenic variants within the *CDKN1C*, *IGF2*, *PLAG1*, and *HMGA2* genes have been described in a minor fraction of SRS patients. To date, the diagnosis remains clinically based in about 40% of SRS patients (Wakeling et al., 2017; Abi Habib et al., 2018).

Among the “non-cardinal” clinical features of SRS, Isolated LO Myoclonus-Dystonia (M-D) has been reported in six SRS patients, all carrying maternal uniparental disomy of chromosome 7 (UPD(7)mat) (Guettard et al., 2008; El-Shewy and Luttrell, 2009; Stark et al., 2010; Sheridan et al., 2013; Martins et al., 2020). M-D is a rare movement disorder with onset in the first 2 decades of life, characterized by truncal and upper-limb myoclonus associated with cervical dystonia and/or writer’s cramp. Depression, panic attacks, and obsessive–compulsive disorders are also included in the non-motor symptoms (Peall et al., 2011). Loss-of-function mutations in *SGCE* (7q21.3) have been identified in 20% of M-D patients (Peall et al., 2014), where the disorder shows autosomal dominant inheritance with reduced penetrance when transmitted by the mother, with the gene being maternally imprinted. This finding led to the hypothesis that the lack of gene expression in UPD(7)mat SRS patients exhibiting M-D might be the driver mechanism of the neurological manifestation. However, the prevalence of M-D in SRS cohorts with UPD(7)mat is low (Guettard et al., 2008; Sheridan et al., 2013; Lokulo-Sodipe et al., 2020; Martins et al., 2020). To our knowledge, unlike UPD(7)mat SRS patients, SRS patients exhibiting dystonia due to hypomethylation of the paternal allele at *H19/IGF2:IG-DMR* have not yet been described.

Here, we report the clinical history of a patient referred for apparently Isolated Lateralized Overgrowth (ILO) and molecularly diagnosed with SRS. Interestingly, since her 16th birthday, our SRS girl has displayed symptoms suggestive of hand dystonia.

## 2 Materials and methods

In accordance with the manufacturer’s protocols, DNA was extracted from peripheral blood lymphocytes (automated extractor, Tecan Group Ltd.) of sibs and parents, from the proband’s epithelial buccal cells (Oragene tubes OG-575 and DNA kit, DNA Genotek Inc.), and from proband’s urine samples (Wizard Genomic DNA Purification Kit, Promega).

The MRC-Holland kit (MRC Holland, Amsterdam, Netherlands) ME-030 BWS/RSS C3-1,017 was used for the molecular diagnosis, in accordance with the kit instructions. Raw data were analyzed by using Coffalyser.Net software (version 140,701- MRC Holland).

Whole-exome sequencing (WES) was performed on genomic DNA from proband and parents (trio) at BIODIVERSA srl service (Milan, Italy) using the SureSelect Human All Exon V7 library and the Illumina HiSeq X platform. Bioinformatic analyses were carried out at the Bioinformatics Unit of the IRCCS Istituto Auxologico Italiano (Milano, Italy). FASTQ data were aligned to the reference genome assembly GRCh37/hg19 using the maximal exact matches algorithm in the Burrows–Wheeler Aligner (BWA) (v.0.7.10). PCR duplicate removal was performed by using Picard (v1.119) (picard.sourceforge.net/). UnifiedGenotyper (GATK v3.7) was used to locally realign insertion/deletions (InDels) and recalibrate base quality scores. Variants were visually inspected using the Integrative Genomics Viewer (IGV, Broad Institute) and further annotated using wANNOVAR (http://wannovar.wglab.org/). Variants with a coverage of >20x and a variant allele frequency of more than or equal to 0.35 were selected for quality filtering. Next, we designed the following two virtual panels of genes to scrutinize putative variants: one related to overgrowth syndromes and the other to dystonia (Supplementary Table S1). Variants were filtered by minor allele frequency (MAF) < 1% in the 1,000 Genomes, Genome Aggregation Databases (gnomAD), and Exome Aggregation Consortium (ExAC) databases. *In silico* prediction of missense variants’ pathogenicity was performed by combining the PolyPhen-2, SIFT, and CADD algorithms. The interpretation of the variants was based on the classification by the VarSome and InterVar databases (Li and Wang, 2017; Kopanos et al., 2019) in accordance with the ACMG–AMP (American College of Medical Genetics and Genomics/Association for Molecular Pathology) guidelines (Richards et al., 2015; Rehder et al., 2021). Selected variants were validated by Sanger sequencing.

Array CGH analysis was performed using the high-resolution Human Genome CGH Microarray Kits 2 × 400K (median resolution of 16 kb) (Agilent Technologies) in accordance with the manufacturer’s instructions. Data were analyzed using Agilent CytoGenomics 3.0. Detected copy number variations (CNVs) were interpreted in accordance with the work of Crippa et al. (2019).

## 3 Results

### 3.1 Longitudinal clinical report

**FIGURE 1:**
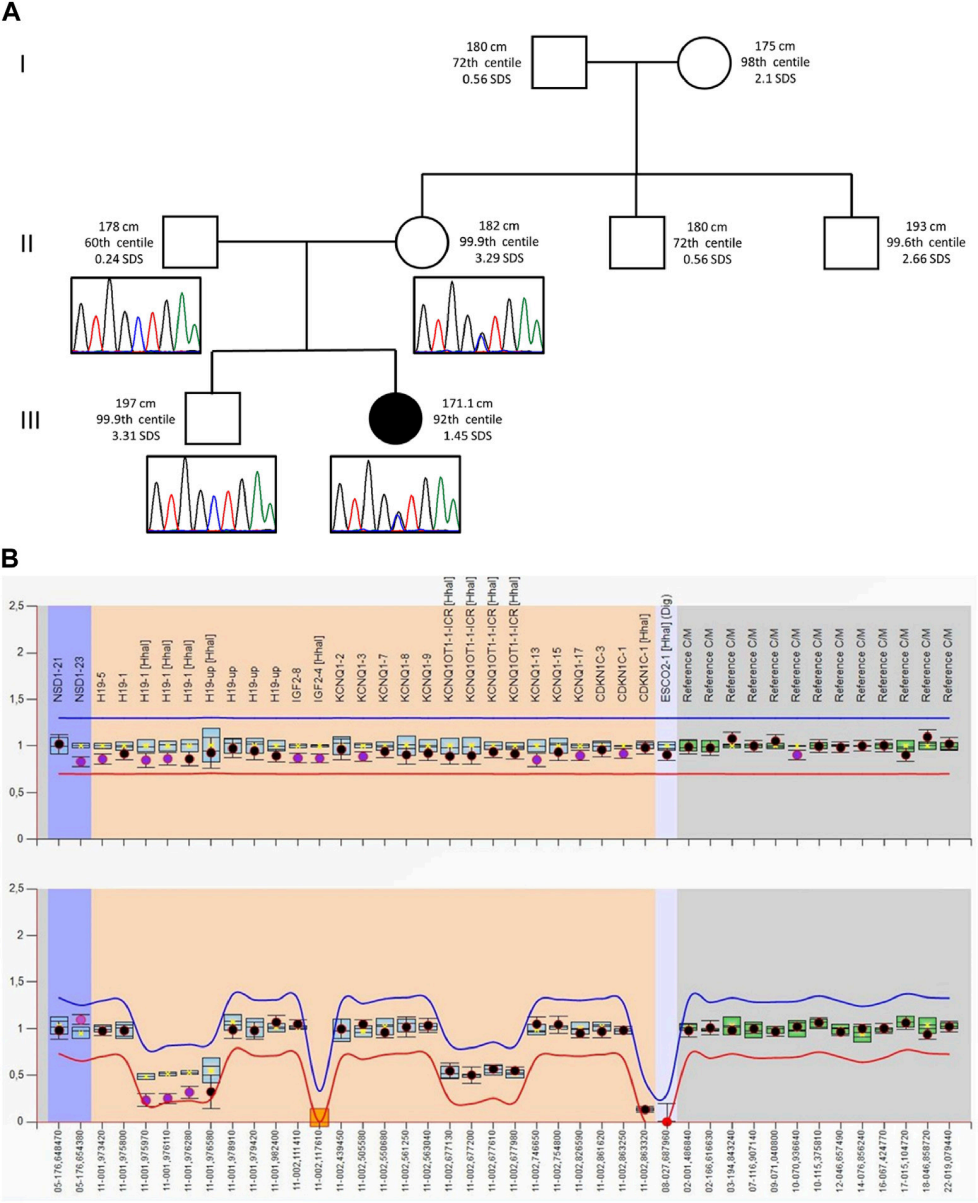
**(A)** Pedigree of the proband’s family: For each member, from the maternal side, the height (cm) and the respective centile and Z-score (SDS) are reported. Height 0–2 years: Who 0–5 years, 2006; height >2 years: Cacciari 2–20 years, Italy, 2006. Electropherograms of the *VPS16* sequence are reported below each correspondent subject. **(B)** MS-MLPA plot of the SRS patient. Analysis of the BWS/RSS MS-MLPA (ME030) shows normal results for the copy numbers (top panel) and a hypomethylation methylation at the *H19* locus (bottom panel).

The proband was second born to unrelated wealthy parents of Caucasian origin living in a small village in the province of Brescia, Northern Italy. Her father and elder brother suffer from depression. Figure 1 shows that her paternal and, especially, maternal family members exhibit tall stature. After 37 weeks of gestation, a cesarean section was performed because of intrauterine growth retardation (IUGR). Her birth weight was 2,310 g (−1.57 SDS), birth length was 46 cm (−1.11 SDS), and occipitofrontal circumference was (OFC) 53.5 cm (+0.08 SDS). Bilateral talipes equinovarus and left hip dysplasia were recorded. At the age of 2 months, she was referred to a clinical geneticist because of body asymmetry, with a predominance of the right side, involving the face, tongue, chest, and upper and lower limbs. Abdominal and renal ultrasounds were carried out, showing normal findings. The karyotype on peripheral blood lymphocytes was 46,XX. She was diagnosed with Isolated Lateralized Overgrowth (ILO) and started a follow-up composed of periodic abdomen ultrasound, orthopedic evaluation, and alpha-fetoprotein test. Developmental milestones were achieved at the expected age. During childhood, she developed kyphoscoliosis. At the age of 13, she underwent right tibial epiphysiodesis due to a worsening of lower limb asymmetry; 1 year later, the staples had to be removed because of rejection. Bone age has always been delayed by 1 year compared to chronological age. Growth parameters were apparently within the normal standard ranges.

At the age of 16, she has been suffering from episodes of sweating, tremors, and shortness of breath; CBC, thyroid function, blood glucose, and cortisol levels have been repeatedly assessed with normal findings. Furthermore, she started to complain of pain involving the ulnar side of the right hand and wrist, spreading to the forearm, arm, and shoulder, worsening during activity (writing and drawing), and leading to hand disuse, to the point that she was left-handed at the age of 19, at the clinical genetics re-evaluation. Indeed, the inability to use her right hand was a source of major stress for her, as she was studying to become a fashion designer. Several investigations were performed, including X-rays of the right arm and forearm, MRI of the cervical column, brachial plexus, right shoulder and arm, and ultrasound and EMG of the right median nerve. However, no cause has yet been found. The dystonia-specific writer’s type, also referred to as writer’s cramp, has been hypothesized (Sadnicka et al., 2016). Various therapies were employed (iontophoresis, oral intake of steroids Deltacortene 7.5 mg/day for 7 days, then 5 mg/day for 10 days, and 2.5 mg/day for 13 days and vitamin B12–Nicetile 1 tablet a day for 30 days, or antiepileptic pregabalin, and NSAIDs as needed) without any effect. In the last neurological examination, carried out at the age of 23, a slight weakness in the right hand finger V abductor and in the ulnar flexion of the right wrist was observed. The examination appeared otherwise normal, including the absence of a sensitivity deficit in the upper limbs and the absence of the appearance of dystonic aspects during graphic tests (writing and drawing). Based on the hypothesis of focal dystonia without motor symptoms (Peall et al., 2015; Sadnicka et al., 2016), a brain MRI with hemosiderin sequences, which the patient has not yet undergone, was recommended. Furthermore, at the age of 18, she was diagnosed with depressive disorder and treated successfully with benzodiazepines and selective serotonin reuptake inhibitors (SSRIs). At the age of 19, she has been suffering from bipolar mood disorder, which is well controlled with lithium salts. Current therapy involves the oral intake of a benzodiazepine as an anxiolytic drug (alprazolam, 15 drops three times a day), an SSRI antidepressant (escitalopram, 1 tablet in the morning), another antidepressant (trazodone, 1 tablet in the evening), and quetiapine (1 tablet of 100 mg in the evening), as well as lithium (1 tablet of 83 mg in the evening) as antipsychotic drugs.

Despite her behavioral hand dystonia and psychiatric diagnosis, she managed to continue her studies at the university. She particularly cares about her appearance.

### 3.2 Molecular analysis and clinical reassessment

Following the ILO diagnosis, the patient underwent a genetic test for the Beckwith–Wiedemann spectrum (BWSp) to confirm the clinical diagnosis. Methylation analysis at *H19/IGF2:IG-DMR* (IC1) and *KCNQ1OT1:TSS-DMR* (IC2) by MS-MLPA showed IC1_LoM in a mosaic pattern, which is consistent with SRS diagnosis. The same result was obtained by MS-MLPA on DNA from buccal swabs and urinary sediment samples. Furthermore, considering the atypical phenotype for SRS diagnosis and the co-occurrence of IC1_LoM with pathogenic CNVs and/or SNVs in genes associated with overgrowth syndromes, high-resolution array CGH and WES on DNA from peripheral blood were performed.

As the tests did not reveal any candidate CNVs or SNVs, the patient’s clinical history was revised in accordance with the SRS consensus statement (Wakeling et al., 2017). Growth parameters from clinical records are reported in Table 1. At the time of clinical re-evaluation, the patient was 19 years old with a height of 171.1 cm (+1.45 SDS), with a mid-parental height (MPH) of +1.91 SDS (Figure 1), a BMI of 17.3 kg/m2 (−1.80 SDS), and an OFC of 55 cm (+0.27 SDS). It is noteworthy that at the age of 2 years and 4 months, her growth was reported to be normal, although the body mass index (BMI) was −2.29 SDS and the height was −2 SDS below her target (Table 1); however, between the age of 2 years and 10 months and 17 years and 8 months, a catch-up occurred and her final height fits with the MPH. Furthermore, she displays asymmetry of the face, including ears and incisor teeth, causing left deviation of the nose, mouth, and tongue; her left ear is low-settled and posteriorly rotated. Asymmetry also involves the upper and lower limbs, with a leg length discrepancy of 0.5 cm after orthopedic surgery. She shows bilateral blepharoptosis and ligamentous laxity with a negative Beighton score. Table 2 shows that the patient exhibited three out of six NH-CSS diagnostic criteria.

**TABLE 1:** Growth parameters of the proband at different ages. Female growth reference charts are provided in the footnote.

Age	OFC, cm (SDS)	Height, cm (SDS)	Weight, kg (SDS)	BMI, kg/m^2^(SDS)
Birth (37 weeks)	33.5 (0.08)	46.0 (−1.11)	2.310 (−1.57)	10.92 (−2.17)
2 m	38.5 (−0.58)	na	4.120 (−2.71)	na
1 year	44.0 (−0.70)	73.5 (−0.28)	7.0 (−2.09)	12.96 (−2.75)
2y 4m	NA	90.0 (−0.09)	10.5 (−1.92)	12.96 (−2.29)
2y 10m	NA	96.0 (0.35)	11.3 (−1.94)	12.26 (−3.49)
17y 8m	NA	170.5 (1.36)	52.7 (−0.40)	18.13 (−1.35)
18y 11m	55.0 (0.27)	171.1 (1.45)	50.9 (−0.66)	17.39 (−1.80)

Birth measurements: INeS charts, Italy, 2010.

OFC, 0–2 years: WHO 0–5 years, Italy, 2006; OFC > 2 years: Hall et al., handbook of physical measurements, 2nd edition, Oxford University Press, 2006.

Height, 0–2 years: WHO 0–5 years, 2006; height > 2 years: Cacciari et al. (2006).

Weight, 0–2 years: WHO 0–5 years, 2007; weight > 2 years: Cacciari et al. (2006).

BMI, 0–2 years: WHO 0–5 years, 2006; BMI > 2 years: Cacciari et al. (2006).

**TABLE 2:** NH-CSS criteria for clinical SRS/SRS-like diagnosis exhibited by the patient.

NH-CSS criteria Wakeling et al. (2017)	Patient’s features	Present/absent
SGA (birth weight and/or birth length ≤ -2 SDS)	Birth weight = −1.57 SDS	Absent
	Birth length: −1.11 SDS	
Postnatal growth failure (height at 24 ± 1 month ≤ -2 SDS or height ≤ -2 SDS below mid-parental height)	Height at 28 months = −0.09 SDS	Absent
	Current height = 1.45 SDS and TH = 1.91 SDS	
	Delta SDS TH = −0.46	
Relative macrocephaly at birth (head circumference at birth ≥ 1.5 SDS above birth weight and/or length SDS)	Birth weight = −1.57 SDS	Present
	Head circumference = 0.08 SDS	
	Delta SDS = 1.65	
Protruding forehead as a toddler (1–3 years)	Absent according to clinical records and the mother	Likely absent
Body asymmetry (LLD of ≥ 0.5 cm of arm asymmetry or LLD of <0.5 cm with at least two other asymmetrical body parts (one non-face)	Asymmetry of face, UL, and LL	Present
Feeding difficulties and/or low BMI (BMI ≤ -2 SDS at 24 months or current use of feeding tube or cyproheptadine)	BMI at 26 months = −2.63 SDS	Present
NH-CSS total score		3/6

### 3.3 Investigating a genetic cause of dystonia

With the aim of revealing pathogenic variants explaining the neurological phenotype, we also interrogated WES for a wide panel of genes involved in the dystonia phenotype (Supplementary Table S1). The only interesting variant identified was the missense one (NM_022575.4:c.2185C>G:p.Leu729Val) in exon 22 of the *VPS16* (vacuolar protein-sorting 16 homolog) gene. This variant has never been reported in 1,000 Genomes, ExAC, or gnomAD databases; it is *in silico* predicted to be damaging (CADD = 23.9; Polyphen-2 = 0.95; and SIFT = 0.005), and the aminoacid Leu729 is evolutionarily conserved. Using the ACMG criteria, we classified it as a variant of uncertain significance (PM1, PM2, PP3, and BP1). Variants in the *VPS16* gene are associated with Dystonia 30 (DYT30, MIM#619291). The segregation analysis showed that the variant was inherited from the asymptomatic mother and it is absent in her brother.

## 4 Discussion and conclusion

Silver–Russell syndrome is a heterogeneous clinical and genetic condition, mainly characterized by lower growth parameters than the population. The growth rate and the final height in adulthood are highly heritable continuous quantitative traits. A number of genetic, hormonal, environmental, and nutritional factors can concur to influence the final phenotype. Given the complex etiology of this trait, suspicion of growth retardation is particularly difficult (Gudbjartsson et al., 2008). Here, we describe a patient initially diagnosed as BWSp by her ILO, in accordance with the diagnostic consensus of the syndrome (Brioude et al., 2018). Despite asymmetric body growth being one of the six specific clinical criteria listed in the NH-CSS for SRS (Wakeling et al., 2017), our patient’s growth parameters at birth and postnatally did not raise the suspicion of SRS (Table 1). However, her growth chart was below the familial target, since her mid-parental target height is higher than the reference one in the population. To our knowledge, few SRS patients have been reported with normal growth parameters (height > −2 SDS) (Bliek et al., 2006; Zeschnigk et al., 2008; Russo et al., 2016; Patti et al., 2018; Mackay et al., 2019; Lokulo-Sodipe et al., 2020), and only two patients have been described as having a final height of about 170 cm without receiving growth hormone treatment (Bliek et al., 2006; Lokulo-Sodipe et al., 2020). However, in both studies, parental height was not indicated. The patient reported by Bliek and others differed from our case for SGA (the birth length was unknown) and feeding difficulties, while that reported by Lokulo-Sodipe et al. (2020) shares two NH-CSS diagnostic criteria with our case. This suggests that, in evaluating patients with an ILO phenotype without marked growth delay, the presence of NH-CSS criteria (such as relative macrocephaly) and the mid-parental height should be considered, as the genetic background can influence the syndrome’s phenotypic expression.

**FIGURE 2:**
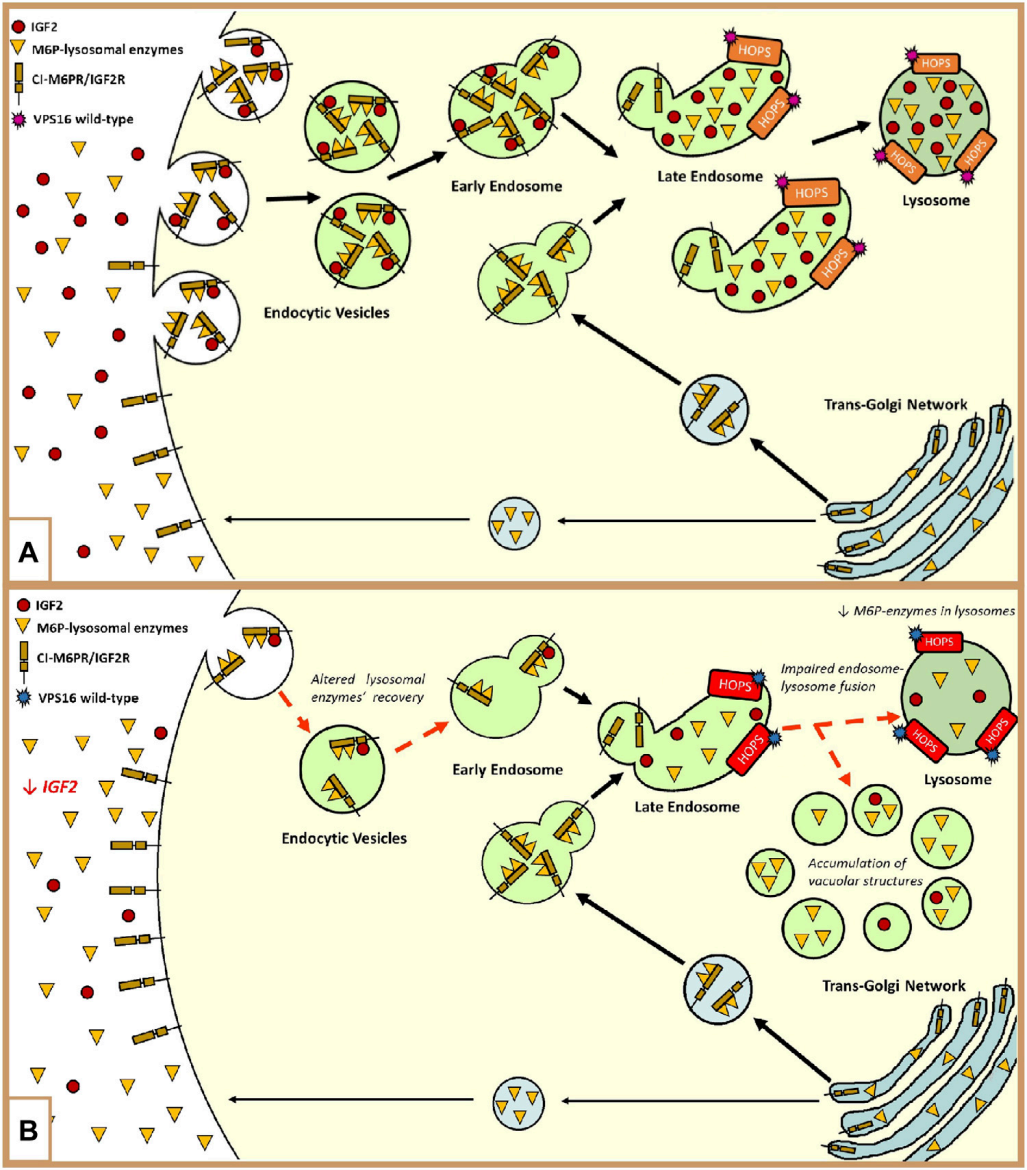
**(A)** Representation of the involvement of IGF2 and the HOPS complex in the endolysosomal pathway: The lysosomal enzymes are synthesized in the trans-Golgi network and delivered to the endolysosomal system through the Golgi cation-independent mannose-6-phosphate receptor (CI-M6PR/IGF2R). During trafficking, some proteins escape binding with the receptor, and they are exocytosed into the extracellular compartment (Bajaj et al., 2019). These enzymes can be retrieved and rerouted back to the endolysosomal system by the cell surface CI-M6PR/IGF2R. The recovery of these enzymes is modulated by the binding of extracellular IGF2 in an enzyme type-dependent manner. In both ways, lysosome enzymes reach their final destination by means of the fusion of late endosomes to lysosomes mediated by the HOPS complex, to which VPS16 belongs (El-Shewy and Luttrell, 2009; Wang et al., 2017; Monfrini et al., 2021). Furthermore, the endocytosis of extracellular IGF2 is important for its degradation and the regulation of its bioavailability (Oka et al., 1985; Olson et al., 2020; Braulke et al., 1990; El-Shewy and Luttrell, 2009; Zavorka et al., 2016; Wang et al., 2020). **(B)** Hypothetical dysregulation of the endolysosomal system in our patient: The loss of methylation at IC1 leads to low extracellular levels of IGF2. In this context, the recovery of the escaped M6P-lysosomal enzymes and IGF2 is impaired. The maturation of the endocytic vesicles, containing lysosomal enzymes, proceeds until the state of late endosome. At this level, the presence of the *VPS16* variant slightly affects the activity of the HOPS complex in promoting the fusion between the late endosome and lysosome. This leads to an abnormal amount of M6P enzymes in the lysosomes and to the formation and accumulation of vacuolar structures, containing these enzymes, in the cytoplasm (Monfrini et al., 2021).

Moreover, in the distinctive growth chart, our patient shows a neurological disorder, manifesting hand-writer’s cramp, and psychiatric disorders such as bipolar disorder and depression, which may be part of an M-D’s phenotype (Peall et al., 2011). To our knowledge, this patient is the first SRS exhibiting this disorder, associated with hypomethylation of the paternal allele at *H19/IGF2:IG-DMR,* and we hypothesized that an additional hit might be involved. WES analysis did not reveal pathogenic variants in candidate genes previously associated with M-D; out of the few identified VUSs (Supplementary Table S2), we focused on those with a putative interaction with the *IGF2* pathway. Specifically, we focused on the *VPS16* variant (p.Leu729Val). The *VPS16* gene encodes a protein belonging to the HOPS (homotypic fusion and protein sorting) complex, a structural bridge composed of four Vps-C core proteins (VPS11, VPS16, VPS18, and VPS33A), and two additional subunits (VPS39 and VPS41), which are necessary for the fusion of late endosomes and autophagosomes with the lysosomes in the cytoplasm (Luzio et al., 2007; Ostrowicz et al., 2010; Monfrini et al., 2021). Figure 2A summarizes the connection between the degradation of IGF2 and the recovery of extracellular M6P-lysosomal enzymes through the plasma membrane cation-independent mannose-6-phosphate receptor (CI-M6PR/IGF2R) (Oka et al., 1985; Braulke et al., 1990; El-Shewy and Luttrell, 2009; Zavorka et al., 2016; Wang et al., 2017; 2020; Bajaj et al., 2019). Dysregulation of the endolysosomal and autophagic systems is linked to the pathogenesis of dystonia, which is a feature of the clinical presentation of many lysosomal storage disorders (Monfrini et al., 2021).

Several heterozygous *VPS16* variants (17 loss-of-function and three missense variants, Supplementary Table S3) were found to co-segregate with dystonia in multigenerational families displaying a dominant pattern of inheritance with incomplete penetrance and wide clinical expressivity of the age of onset and dystonia severity (Steel et al., 2020; Ostrozovicova et al., 2021; Park et al., 2022. Patients with heterozygous VPS16 variants often display psychiatric features such as depression or bipolar disorder (Monfrini et al., 2021). Our patient fits the clinical phenotype of the reported *VPS16*-mutated patients and the segregation of their variants (overviewed in Supplementary Table S4). Clinical data were collected from all 32 heterozygous *VPS16* patients described in the literature. Many patients displayed early-onset dystonia (0–20 years, 25/32), and the most frequent first symptom was isolated writer’s cramp (10/32) (Cai et al., 2016; Steel et al., 2020; Li L.-X. et al., 2021; Li X. et al., 2021; Ostrozovicova et al., 2021; Pott et al., 2021; Gu et al., 2022; Park et al., 2022; Petry-Schmelzer et al., 2022).

Despite few missense variants in the C-terminal domain of VPS16 being reported, *in vitro* mutagenesis experiments (Ala669Asp and Leu725Glu) support the relevant role of the HOPS domain by showing that variants in this domain may severely affect the VPS16-VPS33A binding and mutations proximal to the interaction surface could interfere in the binding (Graham et al., 2013). Furthermore, Park et al. identified 11 missense variants in a wide dystonic cohort. Although missense variants were not enriched in this cohort compared to non-dystonic patients, the authors did not exclude their roles in the disease, or other genetic, epigenetic, and environmental factors that may contribute to disease manifestation and severity (Park et al., 2022). Otherwise, since no other patients affected by dystonia have so far been reported with the VPS16 (NM_022575.4):c.2185C>G:p.Leu729Val, the proband’s hand dystonia might be one of the rare clinical phenotypes of the SRS spectrum, whose pathomechanism still needs to be elucidated. Based on this hypothesis, the patient’s psychiatric manifestations might be interpreted as a more severe manifestation of familial depressive disorder.

To sum up, we hypothesize that in our proband, the low extracellular levels of IGF2, due to the IC1_LoM, may worsen the effect of the co-present *VPS16* variant and contribute to the expression of the neurological phenotype (Figure 2B).

Discrepant phenotypes have been reported for SRS and BWSp (Mackay et al., 2019), but confounding factors such as mid-parental height should be considered, as well as the presence of the diagnostic criteria of either syndrome. The path to the molecular diagnosis in our patient has been tortuous, and recognition of SRS came late. It is true to say that the result of the genetic test in childhood could have led to clinical diagnosis and the proper follow-up. Indeed, as an adult, the patient has expressed disappointment about the long-lasting and ineffective follow-up for the timely diagnosis of embryonic tumors. On the other hand, SRS patients are susceptible to developing metabolic syndrome in adulthood (Patti et al., 2018), whereas BWS/ILO patients can interrupt the follow-up. Interestingly, the deficit of *IGF2* may correspond to the expression of a *VPS16*-related dystonia phenotype.

The description and discussion of this unique SRS patient, in addition to being useful in the clinical setting to reach the proper diagnosis and management, may enhance the understanding of the molecular basis of the broad SRS spectrum.

## Data Availability

The datasets presented in this study can be found in online repositories. The names of the repository/repositories and accession number(s) can be found in the article/Supplementary Material. https://doi.org/10.7910/DVN/JBDF2Q.
