# Erratum: Case report: atypical Silver-Russell syndrome patient with hand dystonia: the valuable support of the consensus statement to the wide syndromic spectrum

**DOI:** 10.3389/fgene.2024.1372019

**Published:** 2024-01-25

**Authors:** Frontiers Production Office

**Affiliations:** Frontiers Media SA, Lausanne, Switzerland

**Keywords:** Silver-Russell syndrome, unexpected molecular results, body asymmetry, IGF2, hand dystonia, VPS16, endolysosomal pathway

Due to a production error, the **title** of the original article was incorrectly modified, using “*Case report: a typical Silver-Russell syndrome patient with hand dystonia: the valuable support of the consensus statement to the wide syndromic spectrum*” instead of “*Case report: atypical Silver-Russell syndrome patient with hand dystonia: the valuable support of the consensus statement to the wide syndromic spectrum*”.

The publisher apologizes for this mistake. The original version of this article has been updated.

