# Supplementary material for: Case report: atypical Silver-Russell syndrome patient with hand dystonia: the valuable support of the consensus statement to the wide syndromic spectrum
Source: Front Genet. 2023 Jul 17;14:1198821. doi: 10.3389/fgene.2023.1198821 (PMC10387531; doi:10.3389/fgene.2023.1198821)
Supplement: Supplementary file 1 [file DataSheet1.docx]

Supplementary Material

**Case Report: Atypical Silver-Russell syndrome patient with hand dystonia: the valuable support of the consensus statement to the wide syndromic spectrum**

**Alessandro Vimercati^1^, Pierpaola Tannorella^1^, Eleonora Orlandini^2^, Luciano Calzari^1^, Mirella Moro^3^, Sara Guzzetti^1^, Angelo Selicorni^4^, Milena Crippa^1^, Lidia Larizza^1^, Maria Teresa Bonati^5^*, Silvia Russo^1^***

^1^Research Laboratory of Medical Cytogenetics and Molecular Genetics, IRCCS Istituto Auxologico Italiano, Milan, Italy

^2^Specialty School of Pediatrics, Alma Mater University of Bologna, Bologna, Italy

^3^Department of Endocrine and Metabolic Diseases &, Lab of Endocrine and Metabolic Research, IRCCS Istituto Auxologico Italiano, Milan, Italy

^4^UOC Pediatria, ASST Lariana, 22100 Como, Italy

^5^ Unit of Medical Genetics, Institute for Maternal and Child Health Istituto di Ricovero e Cura a Carattere Scientifico (IRCCS) Burlo Garofalo, Trieste, Italy

*** Correspondence:**Maria Teresa Bonati
mariateresa.bonati@burlo.trieste.it

Silvia Russo
[s.russo@auxologico.it](mailto:s.russo@auxologico.it)

| *ACOX1* | *CLN6* | *FASTKD2* | *KIF1C* | *NDUFS8* | *PTS* | *TOR1AIP1* |
| --- | --- | --- | --- | --- | --- | --- |
| *ACSF3* | *CLPB* | *FBXL4* | *KMT2B* | *NDUFV1* | *QDPR* | *TPI1* |
| *ACTB* | *CNBP* | *FBXO7* | *L2HGDH* | *NDUFV2* | *RNASEH2A* | *TPK1* |
| *ADAR* | *COASY* | *FITM2* | *LIPT1* | *NKX2-1* | *RNASEH2B* | *TPP1* |
| *ADCY5* | *COL6A3* | *FKBP5* | *LRRK2* | *NKX6-2* | *RNASEH2C* | *TRAPPC11* |
| *AFG3L2* | *COQ9* | *FOXG1* | *MAPT* | *NPC1* | *RNASET2* | *TREM2* |
| *AIMP1* | *COX20* | *FOXRED1* | *MARS2* | *NPC2* | *SAMHD1* | *TREX1* |
| *ALDH5A1* | *CP* | *FTL* | *MAT1A* | *NUBPL* | *SCN8A* | *TRPM7* |
| *ALDH6A1* | *CSTB* | *FUCA1* | *MCCC1* | *NUP62* | *SCP2* | *TSEN2* |
| *ANO3* | *CTC1* | *GALC* | *MCEE* | *PAH* | *SDHAF1* | *TSFM* |
| *AOPEP* | *CTSD* | *GALT* | *MCOLN1* | *PANK2* | *SEPSECS* | *TTC19* |
| *AP1S2* | *CTSF* | *GAMT* | *MECP2* | *PARK2* | *SERAC1* | *TUBB4A* |
| *AP4B1* | *CYP27A1* | *GATM* | *MECR* | *PARK7* | *SGCE* | *UQCRQ* |
| *APTX* | *D2HGDH* | *GBA* | *MFSD8* | *PCCA* | *SLA* | *VAC14* |
| *ARSA* | *DCAF17* | *GCDH* | *MMAA* | *PCCB* | *SLC13A5* | *VAMP1* |
| *ARX* | *DCTN1* | *GCH1* | *MMAB* | *PCDH12* | *SLC16A2* | *VPS10* |
| *ATCAY* | *DDC* | *GLB1* | *MMADHC* | *PCDH19* | *SLC19A3* | *VPS11* |
| *ATM* | *DHDDS* | *GLRA1* | *MPV17* | *PDC* | *SLC20A2* | *VPS13A* |
| *ATP13A2* | *DLAT* | *GM2A* | *MR1* | *PDGFB* | *SLC2A1* | *VPS13B* |
| *ATP1A2* | *DLD* | *GNAL* | *MRE11* | *PDGFRB* | *SLC30A10* | *VPS13D* |
| *ATP1A3* | *DMPK* | *GNAO1* | *MRE11A* | *PDHA1* | *SLC39A14* | *VPS16* |
| *ATP7B* | *DMXL2* | *GNB1* | *MT-ND6* | *PDHX* | *SLC46A1* | *VPS18* |
| *ATP8A2* | *DNAJC12* | *GOSR2* | *MTO1* | *PINK1* | *SLC6A19* | *VPS3* |
| *ATXN1* | *DNAJC5* | *GRIK2* | *MUT* | *PLA2G6* | *SLC6A3* | *VPS33A* |
| *ATXN2* | *DNAJC6* | *GRN* | *NDUFA1* | *PLP1* | *SLC6A8* | *VPS33B* |
| *ATXN3* | *DRD2* | *HDC* | *NDUFA10* | *PNKD* | *SMPD1* | *VPS35* |
| *ATXN8OS* | *DRD5* | *HEXA* | *NDUFA11* | *PNKD2* | *SPAST* | *VPS37A* |
| *AUH* | *DYT1* | *HIBCH* | *NDUFA12* | *PNKP* | *SPATA5L1* | *VPS39* |
| *B4GALNT1* | *DYT13* | *HPCA* | *NDUFA2* | *PNPLA8* | *SPR* | *VPS41* |
| *BCAP31* | *DYT15* | *HPRT1* | *NDUFA9* | *PNPT1* | *SQSTM1* | *VPS4A* |
| *BTD* | *DYT17* | *HRAS* | *NDUFAF1* | *POLG* | *SUCLA2* | *VPS53* |
| *C19orf12* | *DYT21* | *HTRA2* | *NDUFAF3* | *POLR3A* | *SUCLG1* | *VPS8* |
| *C9orf72* | *DYT23* | *HTT* | *NDUFAF4* | *POLR3B* | *SUOX* | *WDR45* |
| *CA8* | *DYT7* | *JPH3* | *NDUFAF5* | *PPP2R2B* | *SURF1* | *WDR73* |
| *CACNA1A* | *EARS2* | *KCNA1* | *NDUFAF6* | *PPT1* | *SYNJ1* | *XK* |
| *CACNA1B* | *ECHS1* | *KCNJ6* | *NDUFB3* | *PRKCG* | *TAF1* | *XPR1* |
| *CBS* | *EIF2AK2* | *KCNMA1* | *NDUFB9* | *PRKN* | *TBX18* | *YY1* |
| *CHMP2B* | *EKD2* | *KCNN2* | *NDUFS1* | *PRKRA* | *TH* | *ZC4H2* |
| *CIZ1* | *ERCC6* | *KCNQ2* | *NDUFS2* | *PRRT2* | *THAP1* |  |
| *CLN3* | *EXOSC3* | *KCTD17* | *NDUFS6* | *PSEN1* | *TIMM8A* |  |
| *CLN5* | *FA2H* | *KCTD7* | *NDUFS7* | *PTEN* | *TOR1A* |  |

**Supplementary Table 1**. List of dystonia-associated genes.

**Supplementary Table 2.** List of Variants of Uncertain Significance (VUS) disclosed by WES analysis. The classification was made according to ACMG-AMP Guidelines (Richards et al., 2015; Rehder et al., 2021).

AD, autosomal dominant; AR, autosomal recessive

| **Gene** | **Coding DNA level** | **Inheritance** | **Associated disease** | **ACMG classification** |
| --- | --- | --- | --- | --- |
| *HRAS* | NM_176795:  c.142G>T;p.Gly48Trp | Paternal | Costello Syndrome (AD, MIM #218040) | VUS (PM2, PP3) |
| *SPAST* | NM_014946:  c.1297C>T;p.Leu433Phe | Paternal | Spastic paraplegia 4 (MIM #182601) | VUS (PM1, PM2, PP3) |
| *DHDDS* | NM_205861.3:  c.496G>C;p.Val166Leu | Maternal | Developmental delay and seizures with or without movement abnormalities (MIM #617836) | VUS (PM2) |
| *VPS13B* | NM_152564.5:  c.11191T>A;p.Ser3731Thr | Paternal | Cohen Syndrome (MIM #216550) | VUS (PM2, BP1) |

**Supplementary Table 3.** Type and recurrence of *VPS16* variants identified in previous reports.

# The occurrence of the same variant in unrelated patients or family.

| ***VPS16* Variants (NM_022575.3)** | **Number of reports** | **Publications** |
| --- | --- | --- |
| c.133_134dup;p. Pro46Alafs*6 | - | Li L.-X., et al., 2021 |
| c.156C>A;p.Asn52Lys | - | Cai et al., 2016 |
| c.244_259delinsGAGAGC;p.Lys82Glufs*124 | - | Pott et al., 2021 |
| c.436del;p.Ile146Serfs*65 | - | Steel et al., 2020 |
| c.455_462dup;p.Leu155Alafs*59 | - | Steel et al., 2020 |
| c.559C>T;p.Arg187Ter | 4# | Steel et al., 2020;  Ostrozovicova et al., 2021 |
| c.692A>G;p. Tyr231Cys | - | Gu et al., 2021 |
| c.721_727del;p.Gly241Serfs*47 | - | Park et al., 2022 |
| c.1094_1095dup;p.Tyr366Serfs*12 | - | Steel et al., 2020 |
| c.1189A>G;p.Lys397Glu | - | Pott et al., 2021 |
| c.1367+2T>C;p.? | - | Steel et al., 2020 |
| c.1335T>G;p.Tyr455Ter | - | Steel et al., 2020 |
| c.1612-1G>C;p.? | - | Steel et al., 2020 |
| c.1661A>C;p.Lys554Tyr | - | Li XY et al., 2021 |
| c.1720+1G>C;p.? | - | Steel et al., 2020 |
| c.1903C>T;p.Arg635Ter | 5# | Steel et al., 2020; Park et al., 2022;  Petry-Schmelzer et al., 2022 |
| c.1929_1930del;p.Arg643fs* | - | Li et al., 2020 |
| c.1939C>T;p.Arg647Ter | - | Park et al., 2022 |
| c.1988_1989insG;p.Asn663Lysfs*2 | - | Steel et al., 2020 |
| chr20:2835462–3974387 microdeletion | - | Steel et al., 2020 |

**
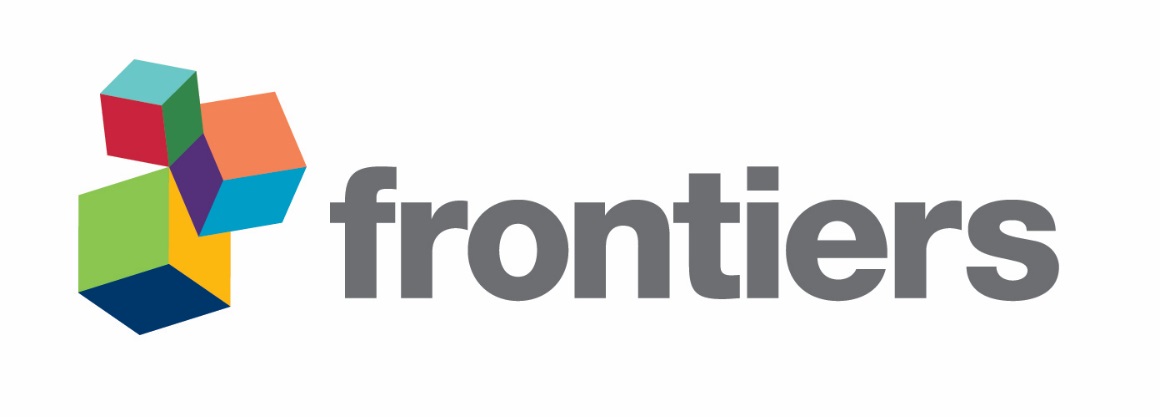
**
